# Motor learning deficits in cervical dystonia point to defective basal ganglia circuitry

**DOI:** 10.1038/s41598-021-86513-7

**Published:** 2021-04-01

**Authors:** Sebastian Loens, Julius Verrel, Vera-Maria Herrmann, Amrei Kienzle, Elinor Tzvi, Anne Weissbach, Johanna Junker, Alexander Münchau, Tobias Bäumer

**Affiliations:** 1 Institute of Systems Motor Science, University of Lübeck, Lübeck, Germany; 2 Department of Neurology, University of Leipzig, Leipzig, Germany; 3 Department of Neurology, University of Lübeck, Lübeck, Germany

**Keywords:** Dystonia, Basal ganglia, Cerebellum

## Abstract

Dystonia is conceptualized as a network disorder involving basal ganglia, thalamus, sensorimotor cortex and the cerebellum. The cerebellum has been implicated in dystonia pathophysiology, but studies testing cerebellar function in dystonia patients have provided equivocal results. This study aimed to further elucidate motor network deficits in cervical dystonia with special interest in the role of the cerebellum. To this end we investigated motor learning tasks, that differ in their dependence on cerebellar and basal ganglia functioning. In 18 cervical dystonia patients and 18 age matched healthy controls we measured implicit motor sequence learning using a 12-item serial reaction time task mostly targeting basal ganglia circuitry and motor adaptation and eyeblink conditioning as markers of cerebellar functioning. ANOVA showed that motor sequence learning was overall impaired in cervical dystonia (p = 0.01). Moreover, unlike healthy controls, patients did not show a learning effect in the first part of the experiment. Visuomotor adaptation and eyeblink conditioning were normal. In conclusion, these data lend support to the notion that motor learning deficits in cervical dystonia relate to basal ganglia-thalamo-cortical loops rather than being a result of defective cerebellar circuitry.

## Introduction

Dystonia is a movement disorder characterized by abnormal involuntary movements often repetitive and patterned^1^. Pathophysiologic concepts of dystonia have evolved from the hypothesis of dystonia representing a prototype basal ganglia disorder to the view of dystonia as a sensorimotor network disorder involving the basal ganglia, cerebellum, thalamus and sensorimotor cortex^2^. Neuro-functionally, dystonia is characterized by reduced inhibition, abnormal sensorimotor integration, maladaptive synaptic plasticity as well as reduced thalamic gating and dysfunctions of thalamo-striatal and cortico-striatal networks^3–6^. Synaptic plasticity is the neuronal mechanism underlying learning processes in general. Motor learning in dystonia patients has shown to be associated with reduced plasticity in inhibitory networks^7^, thus rendering motor learning paradigms within the sensorimotor system a useful tool in dystonia research.

Motor learning is often studied using variants of two main learning paradigms: Motor sequence learning (MSL) and motor adaptation (MA). Theoretical models propose two distinct brain networks to be involved in motor learning: the cerebello-thalamo-cortical (CTC) loop is considered to provide an internal forward model based on sensory states and cortical motor commands, which is then compared to the actual motor outcome. The basal ganglia-thalamo-cortical loop (BGTC) is predominantly engaged in action selection and probabilistic reward-based learning^8,9^. MSL and MA rely on shared cortical and subcortical motor network components involving BGTC and CTC networks, but MA has a greater demand on cerebellar computing^10^. Cerebellar pathology leads to deficits in MSL^11^ and MA^12^, while in patients with predominant basal ganglia pathology, MSL is impaired^13^ but MA is typically not affected^12^. A very different approach to cerebellum-dependent motor learning mechanisms is the investigation of classical eyeblink conditioning, which is typically impaired in patients with cerebellar lesions^14^ and relies basically on cerebellum and brainstem circuits^15,16^.

A special role of the cerebellum in the pathophysiology of dystonia has been a matter of debate^17^. It has been hypothesized that the cerebellum modulates striatal activity and dystonia may arise from disruptions in a cerebellum-basal ganglia network^18,19^. Evidence comes from animal studies^20,21^, and indirectly also from studies of patients with cerebellar lesions^22^. Importantly, dystonia can be a feature of neurodegenerative disorders of the cerebellum^18^. In idiopathic cervical dystonia (CD), both microstructural abnormalities of the cerebellum^23^ and abnormalities in functional activity and connectivity of the cerebellum were found^23^. Reduced cerebellar connectivity has also been implicated by transcranial magnetic stimulation^24^. Previous studies of motor learning designed to assess deficits in cerebellar functioning in dystonia yielded equivocal results. While MA was found normal across different types of dystonia in most (though not all) studies^25–29^, the investigation of eyeblink conditioning found cerebellar learning to be either impaired^30–32^ or normal^33,34^. Abnormal sequence learning was reported in DYT-*TOR1A*^35^, but was found intact in CD^25^ and writer’s cramp^36,37^. However, interpretation of the latter results in the context of involved brain networks is difficult because the applied tasks deviated substantially from the classical serial reaction time task (SRTT)^38^.

This study aimed to examine in detail the deficits in cerebellar-associated motor learning in CD using well established tasks that rely to different extents on the integrity of CTC and BGTC networks. We investigated MSL via the classical SRTT, MA was probed with an established protocol of cerebellum-dependent visuomotor adaptation^39^. Further, eyeblink conditioning was tested, representing a classical cerebellar-learning task that is largely independent of the previously named networks^15,16^.

## Results

### Motor sequence learning

**Figure 1:**
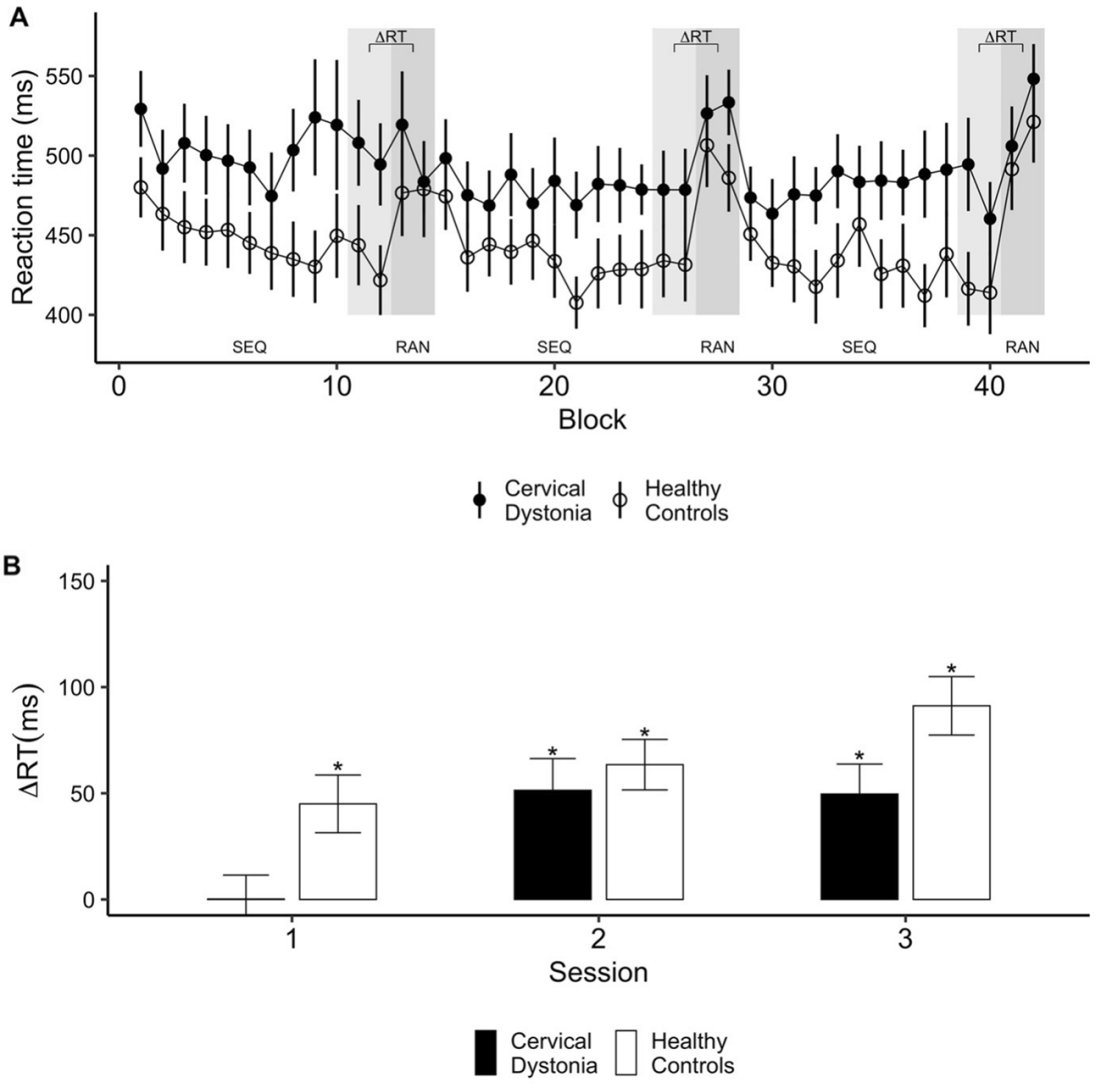
Motor sequence learning. (**A**) Performance in the motor sequence learning task is shown as median reaction time (RT) ± SE for runs of 12 trials. Each block consisted of 12 × 12 sequence trials (SEQ) and 2 × 12 random trials (RAN). Grey bars indicate data points used to characterize sequence learning as ΔRT = RT_RAN_ − RT_SEQ_. (**B**) ANOVA showed reduced sequence learning in cervical dystonia (CD) in general, and pairwise t-tests demonstrated no learning at all in session 1 in CD. *Significant learning (p < 0.05, Bonferroni corrected).

During the SRTT participants had to react to visually presented cues by pressing the corresponding button on the computer keyboard as fast as possible. After an introductory simple task, participants completed three identical sessions comprising 12 repetitions of a 12-item sequence (144 trials) followed by two blocks of 12 pseudo-randomly presented cues each (24 trials). There was a baseline difference in reaction times between groups, i.e. performance of the control group was faster during the simple task (t = 3.53, p < 0.001). Sequence learning was assessed as the reaction time advantage for sequentially repeated (RT_SEQ_) relative to pseudo-randomly presented cues (RT_RAN_), ΔRT = RT_RAN_ − RT_SEQ_ (Fig. 1A), with positive values indicating learning. ANOVA with SESSION (1, 2, 3) as within-subject factor and GROUP as between-subject factor showed a main effect of GROUP (F(1,34) = 7.1, p = 0.01), indicating superior learning in the healthy control (HC) group (ΔRT across all sessions was 66.6 ms ± 7.9 ms (SE) in HC and 33.8 ms ± 8.3 ms in CD patients). A main effect of SESSION (F(2,68) = 8.1, p < 0.001) indicated differences in the amplitude of learning effects between sessions. Post-hoc tests revealed that sequence learning was stronger in session 2 and 3 compared to session 1 (all p < 0.05 Bonferroni corrected). The GROUP X SESSION interaction was not significant.

To further scrutinize these learning effects, we assessed whether ΔRT was statistically different from 0 (i.e., whether RT differed between the sequence and random condition) using Bonferroni corrected paired one-sample t-tests, separately for each group and session. Learning effects were found for all sessions in HC (all p ≤ 0.01, Bonferroni corrected). In contrast, CD patients did not show evidence for learning in session 1 (p = 1) but only in later sessions (session 2 and 3: p < 0.01, see Fig. 1B).

A repeated measures ANOVA of error rates with CONDITION (sequence, random) as within subject factor and GROUP as between subject factor showed no main effect of GROUP, but a significant effect of CONDITION (F(1,34) = 11.1, p = 0.002), reflecting higher error rates in random vs. sequence blocks in both tasks (4% vs. 2%).

### Motor adaptation

**Figure 2:**
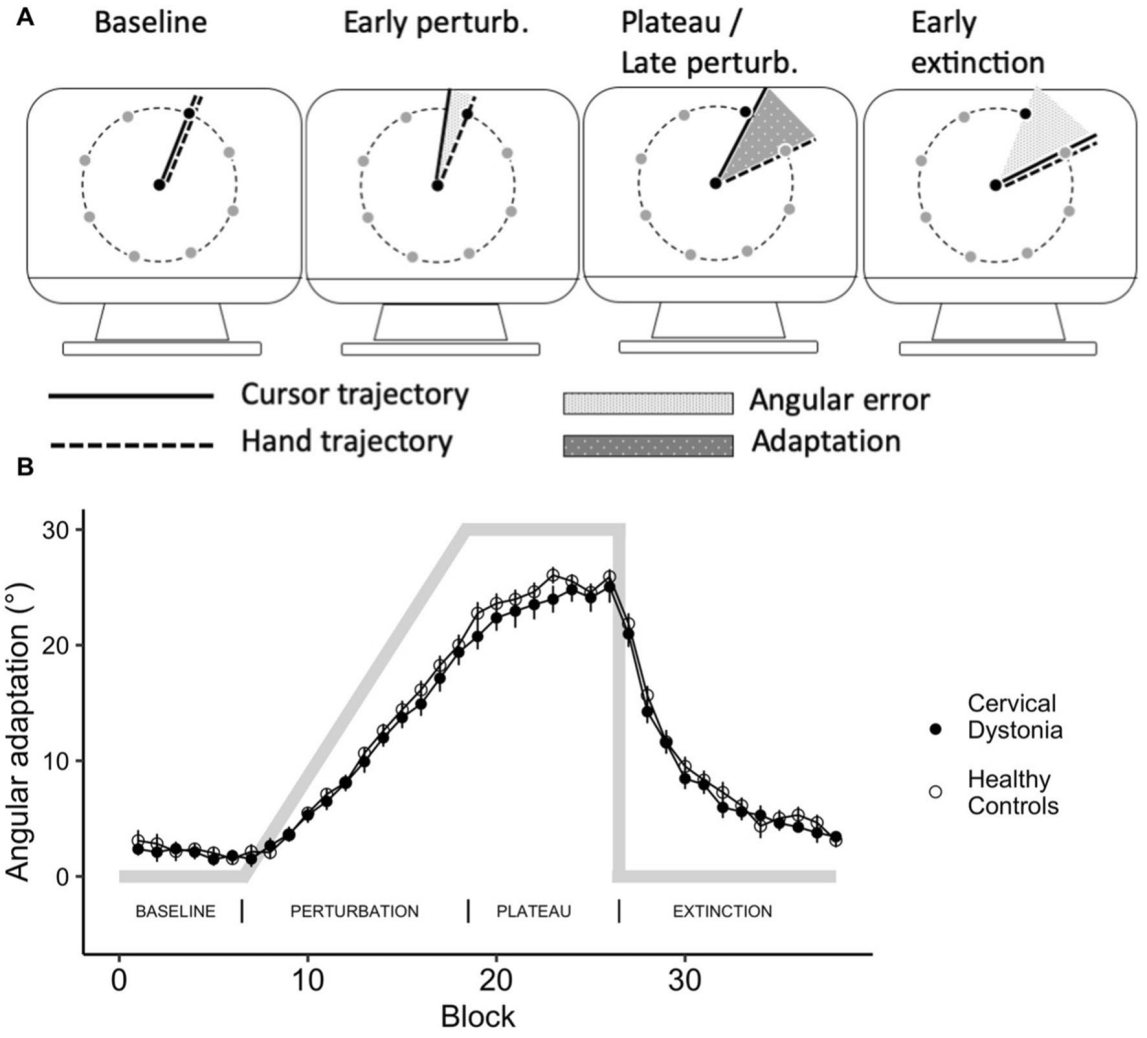
Motor adaptation. (**A**) Schematic course of the experiment indicating difference between visible movements of the cursor on screen and invisible movement of the hand on the digitizing tablet. (**B**) Adaptation to a gradually introduced visuomotor perturbation did not differ between cervical dystonia patients and healthy controls. Points represent adaptation (mean ± SE of eight consecutive trials), grey lines indicate the applied perturbation.

In the visuomotor adaptation task, participants performed fast center-out hand movements to one of eight radially arranged targets. Movements were recorded by a pen on a digitizing tablet, with visual feedback shown on a computer screen. A perturbation of the visual feedback was gradually introduced over 96 trials to a maximum of 30°, maintained for 64 trials (“plateau”) and suddenly removed (“extinction”). Adaptation was measured as the (automatic) adjustment of hand movement trajectories to rotated visuomotor feedback (Fig. 2A). For two CD patients, no data on MA was available. General measures of motor performance (mean movement duration per trial, maximum velocity, maximum pen pressure) were compared with two-sample t-tests and did not differ between groups. To assess MA between groups, we conducted t-tests of the amount of compensatory rotation in degrees at the last two runs of the plateau phase (adaptation; t =  − 0.53, p = 0.61) and at the first two runs of the extinction phase (aftereffect; t =  − 1.05; p = 0.30), indicating that adaptation did not differ systematically between groups (Fig. 2B).

### Classical eyeblink conditioning

**Figure 3:**
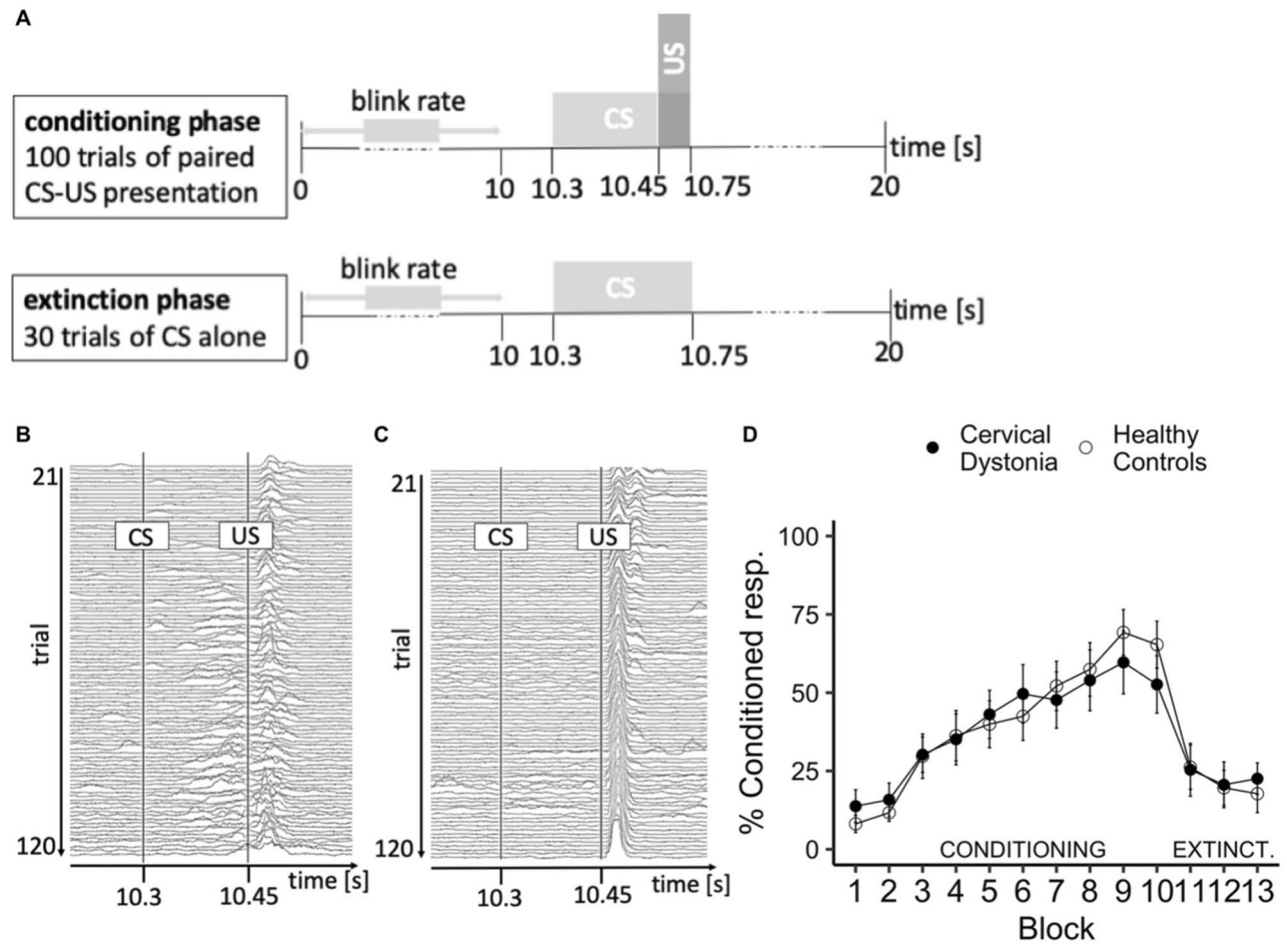
Classical eyeblink conditioning. (**A**) Schematic course of the experiment: In the conditioning phase a tone as the conditioning stimulus (CS; 550 ms, 88 dB), is paired with an air puff as the unconditioned stimulus (US; 100 ms, 110 kPa). In the extinction phase the CS is presented alone. (**B**) Example surface EMG activity of the orbicularis oculi muscle over the course of the conditioning phase of a participant that shows conditioning and (**C**) of a participant that does not exhibit a conditioned response to air puff stimulation. (**D**) Conditioning of the blink reflex is acquired in patients with cervical dystonia to the same extent as in healthy controls. Points represent percentage + SE of conditioned responses of the blink reflex per runs of ten trials.

Eyeblink conditioning refers to pavlovian conditioning of the corneal reflex (Fig. 3A–C). Over ten blocks of ten trials each an air puff was applied to the right eye eliciting a forceful closing of the eyelid. The air puff was preceded by a loud tone as the conditioning stimulus, and conditioning was measured as the percentage of trials per block in which the lid was closed before onset of the air puff. The conditioning phase (100 trials) was followed by an extinction phase (30 trials) in which the tone was presented alone. Repeated measures ANOVA of occurrence of conditioned responses (CR) in the conditioning phase with BLOCK as within-subject factor and GROUP as between-subject factor showed no main effect of GROUP and no interaction, but a significant effect of BLOCK (F(9,306) = 28.6, p < 0.001), indicating that conditioning of the blink reflex to a tone was achieved in both groups, which is reflected by increasing mean values for CR over blocks (Fig. 3D). The same analysis of the extinction phase revealed no main effect of GROUP or BLOCK. The spontaneous blink rate did not differ between groups (HC18/min, CD 25/min, t(29) = 1.4, p = 0.17). To obtain a measure of learning for correlational analysis we collapsed occurrence of CR over block 1 to 5. Mean CR did not differ between groups (t(33) = 0.4, p = 0.68) and did not correlate with the spontaneous blink rate.

### Correlation to clinical parameters and between experiments

Pearson’s correlations with Bonferroni correction for multiple testing did not show any association between measures of motor learning (MSL: mean ΔRT across all sessions; MA: adaptation in degree at the end of plateau; eyeblink conditioning: mean percentage of conditioned responses in block 1–5) and clinical parameters (age at onset, TWSTRS, TRS) or demographic factors (sex, age). Performance measures were not found to be significantly correlated across experiments.

## Discussion

In this study, we compared performance in motor learning tasks involving different networks including the cerebellum between patients with sporadic isolated CD and HC. The main finding of this study is that MSL was impaired in CD patients whereas MA and eyeblink conditioning were normal. Our findings support the view that dystonia is a network disorder involving impaired basal ganglia function. On the other hand, the assumption of a cerebellar dysfunction per se in CD could not be corroborated.

The finding of intact visuomotor adaptation in the MA task agrees with previous studies in CD^25,26^ and writer’s cramp^40^, and also monogenic dystonias such as DYT-*TOR1A*^27^ and DYT-*SGCE*^28^. Also, adaptation of gait during split-belt treadmill walking has been found to be normal in CD, but not in patients with writer’s cramp or blepharospasm^29^. Adaptation to catching balls of different weight has been found to be altered in CD patients with dystonic tremor but not in CD per se^41^. Studies on eyeblink conditioning reported reduced conditioning in CD using a different experimental approach with electrical stimulation of the supraorbital nerve instead of air puff^30^. However, this deficit was later attributed to the presence of dystonic tremor rather than dystonia per se by the same group^33^. In DYT-SGCE, a type of dystonia with myoclonus as the hallmark clinical feature eyeblink conditioning was found to be reduced^32^ or the extinction of eyeblink conditioning was altered^42^ indicating prominent cerebellar dysfunction in these patients and suggesting that myoclonus might be a sign of abnormal cerebellar activity. In patients with DYT-*TOR1A* and DYT-*THAP1* no deficits in eyeblink conditioning have been found^34^. Taken together, evidence from these experiments does not support the assumption of prominent cerebellar dysfunction as a hallmark feature of dystonia per se. Instead, cerebellar abnormalities might rather be related to other clinical features associated with dystonia including tremor or myoclonus in the case of DYT-*SCGE*. Although tremor was present in the majority of dystonia subjects also in this study, tremor severity as measured by the TRS (mean 2.1, range 0–7) was considerably lower compared to studies reporting eyeblink deficits in tremulous dystonia^33^.

MSL deficits in CD patients in this study were present using a 12-item implicit SRTT task, and deficits were most pronounced in the early stages of the experiment. The cerebellar contribution to MSL encompasses formation of an internal model, error reduction, fine tuning of motor components and maintenance of stimulus–response mappings^43^. The proposed role of the basal ganglia is the formation of associations between individual stimuli and movements, i.e. sequence learning^9^. This view has recently been corroborated by a meta-analysis of functional imaging data identifying the anterior striatum and globus pallidus internus (GPi) as the structures responsible for the act of sequence learning^44^. A theoretical model proposes, that learning an implicit sequence can be separated in an early learning phase in which an associative circuit including the anterior-medial striatum and associative parts of the cerebellum, is engaged in in encoding the sequential component of the sequence, resulting in quick reductions of reaction times. Over the course of the experiment activity shifts to sensorimotor circuits, characterized by a shift of neural activity within the striatum to dorso-lateral parts and decreasing cerebellar activity^9^. The finding of a deficit in acquisition of the sequence (reflected by the lack of a sequence vs. random advantage) in the early learning stage might hence be interpreted as an impairment in transition from associative to sensorimotor circuits^9^. In this context it is of special interest that although performance was normal, reduced activity in the anterior putamen and GPi was observed in writer’s cramp patients during MSL^36^. Also, in X-linked dystonia parkinsonism, a hereditary neurodegenerative disease that is characterized by an initial phase of rapidly generalizing dystonia and a later phase dominated by parkinsonism, neuroimaging found degeneration of the anterior putamen and GPi as a hallmark feature of the dystonic phase^45^. It has to be born in mind though that the BGTC and CTC circuits are interconnected through a di-synaptic connection from the dentate nucleus to the striatum^46^, and it has been shown that during early sequence learning the putamen negatively modulates cerebellar activity^47^. The finding of impaired baseline performance in the CD group might indicate abnormal functioning of a sequence learning-independent motor network including the cerebellum^10^. Altered functional and anatomical connectivity between the cerebellum and the basal ganglia has been found in CD and writer’s cramp^23,48^. Moreover, a two-hit model including both the cerebellum and basal ganglia has been proposed for dystonia pathophysiology supported by findings in animal models of dystonia^49^.

The results of the present study contrast earlier findings of intact sequence learning in CD^25^ and writer’s cramp^36^. These discrepancies might result from the use of shorter sequences of five to eight items. Patients with Parkinson’s disease did not show sequence learning in a 12-item sequence, but were able to learn an 8- or 10-item sequence^11^, thus implicating deficits in extracting more and more complex sequences as a result of reduced basal ganglia functioning^13^. On the other hand, MSL was impaired in manifesting and non-manifesting DYT-*TOR1A* (but not DYT-*THAP1*) mutation carriers when learning an 8-item sequence, accompanied by reduced CTC tract integrity^35^. In these patients, dystonia tends to generalize, so that impairments in MSL even when shorter sequences are used might indicate more widespread or profound abnormalities in BGTC and CTC in these patients compared to patients with focal or segmental dystonia as studied here. This is to say that the threshold for MSL deficits to occur may be higher in the latter.

As pointed out above, previous studies indicated that concomitant features such as tremor rather than dystonia per se have been found to be related to deficits in cerebellar dependent learning paradigms^33,41^. The present study was not designed to assess the influence of tremor so that conclusions in this regard are limited. However, although tremor was present in most of our patients tremor rating was usually low. Moreover, we did not find changes in motor learning tasks that were previously associated with tremor^33,41^. We therefore consider it unlikely that our results are confounded by the presence of tremor.

In conclusion, we found deficits in MSL in CD patients whereas motor learning in classical cerebellar learning tasks including MA and eyeblink conditioning was normal. The finding of abnormal basal ganglia related motor learning in cervical dystonia is in keeping with the concept of dystonia as network disorder involving BGTC networks. Although a potential role of the cerebellum in the pathophysiology of dystonia is supported by numerous studies, our findings of deficits in sequence learning do not support the assumption of CD being associated with predominant cerebellar motor control dysfunction.

## Methods

### Participants

For this study we recruited 18 CD patients at least ten weeks after their last botulinum toxin injection and 18 age-matched HC. For only nine CD patients and eight HC data on eyeblink conditioning was available while the remaining either refused participation (dry eyes, use of contact lenses) or showed inconsistent response to the air puff stimulation upon analysis. Hence, another nine patients and ten healthy controls were recruited resulting in 18 participants each (Table 1). Severity of dystonia and tremor was assessed with the Toronto Western Spasmodic Torticollis Rating Scale (TWSTRS) and the Fahn–Tolosa–Marin Tremor Rating Scale (TRS) based on a standardized video protocol. Genetic testing by gene panel analysis has been performed in 20/27 patients. No pathogenic variants were detected in the *TOR1A*, *THAP1*, *GNAL*, *SGCE* or *GCH1* genes.

**Table 1:** Demographics of the study cohort.

	MSL + MA		Eyeblink conditioning	
	CD, N = 18	HC, N = 18	CD, N = 18	HC, N = 18
**Sex, n (%)**				
f	13 (72%)	13 (72%)	13 (72%)	10 (56%)
m	5 (28%)	5 (28%)	5 (28%)	8 (44%)
**Age [years]**				
Mean	61.4	59.6	60.1	61.1
Range	47.0–76.0	46.0–79.0	49.0–68.0	48.0–79.0
**Age at onset [years]**				
Mean	49.2	–	45.6	–
Range	15.0–73.0	–	15.0–65.0	–
**Tremor, n (%)**	14 (78%)	–	13 (72%)	–
**TRS**				
Mean	2.6	–	2.6	–
Range	0.0–6.0	–	0.0–6.0	–
**TWSTRS**				
Mean	13.6	–	16.2	–
Range	6.0–23.0	–	7.0–24.0	–

*CD* cervical dystonia, *HC* healthy controls, *MSL* motor sequence learning, *MA* motor adaptation, *TWSTRS* Toronto Western Spasmodic Torticollis Rating Scale, *TRS* Fahn–Tolosa–Marin Tremor Rating Scale.

The study was approved by the ethics committee of the University of Lübeck (No 17-369). All participants gave written informed consent, and all experiments were performed in accordance to the declaration of Helsinki.

### Serial reaction time task

Four black squares were presented in a horizontal array on a computer screen using Presentation software (Neurobehavioral Systems Inc, Berkeley, USA). When one of the squares turned blue (stimulus), participants were requested to press the corresponding target button on the computer keyboard with the middle or index finger of the left or right hand as quickly as possible. Correct responses were indicated by a change of the stimulus in size and color. Stimuli were presented with a fixed response-stimulus interval of 400 ms. Participants were informed that the items were presented in a repetitive manner but were not told the sequence. Reaction time (RT) was defined as the period from presentation of the stimulus to button presses.

After a practice block that repeated a simple sequence (4–3–2–1–4–3–2–1–4–3–2–1) six times, the main task consisted of three identical sessions, each containing 12 runs of a fixed 12-item sequence (1–2–1–4–2–3-4–1–3–2–4–3) followed by 2 × 12 pseudo-randomly presented stimuli (Fig. 1A).

For statistical analysis, the median RT of each run was calculated. For each session, sequence learning was defined as the difference in mean RT of the last two sequence blocks and the subsequent 2 × 12 pseudo-randomly presented trials, expressed as ΔRT (RT_RAN_ − RT_SEQ_), with positive values indicating a sequence-vs.-random advantage, i.e., learning.

### Motor adaptation

A MA paradigm was programmed in Matlab (The MathWorks, Natick, MA, USA), closely following a previous study^39^. Participants were instructed to draw straight lines from a central starting point “shooting” trough a target in one of eight possible positions arrayed around the starting point at a distance of 70 mm. Targets were equally distributed every 45°. Movements were performed with a hand-held pen on a digitizing tablet (Wacom Intuos Pro L, Wacom, Kazo, Japan) and visualized on a screen. Participants wore special goggles to prevent visual feedback from the moving hand. Targets were presented in pseudo-random order, with each direction appearing once in every block of eight consecutive trials. Over the course of the experiment, a counter-clockwise (visuo-motor) perturbation of the cursor movement displayed on the screen in relation to the actual hand movement trajectory on the tablet was introduced (Fig. 2A).

The cursor was visible during the entire experiment. In case of a hit, the target changed its color to green, otherwise a red dot marked the position where the cursor crossed the (invisible) circle connecting the target positions. When participants hit the target in less than 500 ms, they were rewarded with a pleasant bell sound. If movement durations exceeded 500 ms, a pre-recorded voice (“faster, please!”) was played. Trials with a movement duration > 1000 ms led to immediate repetition of the previous and current trial.

After a practice block, the experiment started with 6 × 8 trials with veridical feedback (baseline condition). In the subsequent perturbation condition, the perturbation was increased in steps of 0.31° degree per trial over 96 (12 × 8) trials to a maximum deviation of 30° degrees counter-clockwise. The perturbation was kept at 30° for another 8 × 8 trials in the plateau condition to allow for further adaptation. Then, in the extinction condition, the perturbation was removed abruptly and kept a veridical feedback over 96 (12 × 8) trials.

In order to minimize the effect of corrective movements, the movement direction was assessed in the “ballistic” phase at 50% of the start-target distance. Adaptation was calculated per trial as the angular difference between the actual movement direction and the respective target angle. For statistical analysis, the mean adaptation per block of eight consecutive trials (i.e., one trial per movement direction) was calculated.

### Classical eyeblink conditioning

Eyeblink conditioning was performed following an established protocol^32^ using air puff stimulation. In conditioning trials, air puffs (100 ms, 110 kPa) as unconditioned stimuli (US) were preceded by a tone (1 kHz, 440 ms) as the conditioning stimulus (CS) (Fig. 3A). After familiarization to the experimental setting (trials 1-20), the conditioning phase started with 10 × 10 conditioning trials (trials 21-120) with paired stimuli (US + CS), followed by 3 × 10 extinction trials (trials 121-150) where the CS was presented alone. A conditioned response (CR) was defined as a blink with onset at least 150 ms after the CS and onset before the US. Blinks were recorded by surface electromyography (EMG) of the orbicularis oculi muscle (Fig. 3B,C). The EMG signals were amplified and filtered (20 Hz and 2 kHz) with a D360 amplifier (Digitimer Limited) and digitized sampled at 5 kHz, digitized using a laboratory interface (Micro 1401; Cambridge Electronics Design, Cambridge, U.K.), and recorded and stored on a personal computer using SIGNAL 6 software (Cambridge Electronic Devices, Cambridge, U.K.). Single trial data were rectified, and smoothed by a low-pass filter (cut-off frequency of 0.06 Hz).

Blinks were automatically identified when having a minimum integral of 0.1 mV x ms and minimum amplitude of 0.001 mV after baseline correction, as well as a minimum duration of 50 ms. Blinks were visually inspected and manually corrected if necessary. Occurrence of CR per block of ten trials was expressed as percentage for further analysis. Trials had a duration of 20 s with onset of the CS after 10.3 s. The spontaneous blink rate was assessed in the first ten seconds of each trial.

### Statistical analysis

Statistical analyses were performed using repeated measures ANOVA or t-test. Post-hoc *t*-tests for statistically significant main effects or interactions were corrected for multiple comparisons (Bonferroni-Holm). Relation of learning between experiments or correlation to clinical parameters was assessed using Pearson’s correlation coefficient. Analysis was conducted in R. A value of p < 0.05 was considered statistically significant.

Original data will be made available upon justified request.
